# The Potential Health Benefits of Noni Juice: A Review of Human Intervention Studies

**DOI:** 10.3390/foods7040058

**Published:** 2018-04-11

**Authors:** Brett J. West, Shixin Deng, Fumiyuki Isami, Akemi Uwaya, Claude Jarakae Jensen

**Affiliations:** 1 Research and Development, Morinda, Inc., 737 East 1180 South, American Fork, UT 84003, USA; shixin_deng@morinda.com (S.D.); jarakae_jensen@morinda.com (C.J.J.); 2 Research and Development, Morinda, Inc., 3-2-2 Nishishinjuku, Shinjuku-ku, Tokyo 160–0023, Japan; fumiyuki_isami@jp.morinda.com (F.I.); akemi_uwaya@jp.morinda.com (A.U.)

**Keywords:** noni juice, *Morinda citrifolia*, clinical trial, antioxidant, immune system, inflammation

## Abstract

Noni juice is a globally popular health beverage originating in the tropics. Traditional Tahitian healers believe the noni plant to be useful for a wide range of maladies, and noni juice consumers throughout the world have similar perceptions. Nevertheless, human clinical trials are necessary for a precise understanding of what the health benefits of noni juice are. A review of published human intervention studies suggests that noni juice may provide protection against tobacco smoke-induced DNA damage, blood lipid and homocysteine elevation as well as systemic inflammation. Human intervention studies also indicate that noni juice may improve joint health, increase physical endurance, increase immune activity, inhibit glycation of proteins, aid weight management, help maintain bone health in women, help maintain normal blood pressure, and improve gum health. Further, these studies point to notable antioxidant activity in noni juice, more so than other fruit juices which served as trial placebos. It is this antioxidant effect and its interaction with the immune system and inflammation pathways that may account for many of the observed health benefits of noni juice. However, the existing evidence does have some limitations as far as its general application to noni juice products; all the peer-reviewed human interventions studies to date have involved only one source of French Polynesian noni juice. Geographical factors and variations in processing methods are known to produce commercial noni juice products with divergent phytochemical and nutrient compositions. Therefore, other sources of noni products may have different toxicological and pharmacological profiles.

## 1. Introduction

Noni juice has become a popular health supplement. In the first dozen years of its commercial marketing, more than 106 million liters of Tahitian Noni^®^ Juice (Morinda, Inc., American Fork, UT, USA) were consumed in more than 80 nations [1]. Noni juice was one of the first whole foods approved under the European Union’s 1997 novel food regulations [2]. The Chinese government has also approved one source of noni juice as a safe new resource and has approved it as a functional food that can enhance immunity [3]. 

Noni is the common name for *Morinda citrifolia*, a small to medium sized tree (3–10 m high) with a pantropical distribution [4]. Noni fruit and leaves have a history of food use among Pacific Islanders as well as in Southern and Southeast Asia. Although the fruit is edible, its flavor has been described as akin to bad cheese [5]. Despite this, Rarotongans ate the fruit often and the Burmese used it to prepare curries [6,7]. Australian Aborigines ate noni fruit during the cool-dry season from May to August in the Northern Territory of Australia [8,9]. Noni leaves were eaten both in raw and cooked form in Java and Thailand [10]. In Tahiti, fish were wrapped in the leaves as a part of baking to impart an appealing flavor to the cooked fish [11].

More recently, the fruit has been used to produce dietary supplements. French Polynesia has been a major source of this juice where noni fruit puree constitutes one of the area’s largest agricultural exports [12]. In fact, more than 21,000 metric tons of this puree was exported in the past decade [13]. The noni plant was the most important and widely used Polynesian medicinal plant prior to the arrival of Europeans with Tahitian healers using it in many remedies [14]. Some uses of the fruit include the treatment of inflammation, abscesses, angina, diabetes, ranula, abdominal fibromas, and scorpionfish stings [15,16]. In other parts of French Polynesia, noni fruit has been reportedly used to treat osteoarthritis, rheumatism, backache, joint problems, hemorrhoids, skin allergies, burns, and warts [17]. The global popularity of the juice is attributable to its perceived health value. Indeed, European consumers of a Tahitian-sourced noni juice beverage frequently have reported health benefits including increased energy, improved well-being, reduction of pain, fewer infections, improved sleep, improved digestion, as well as a reduction in allergy and asthma symptoms [18].

Published research has explored the potential health effects of the noni plant. Unfortunately, much of this research has involved in vitro and animal studies, often using only noni plant extracts or noni juice of unknown concentration [19,20]. Although the findings of these types of studies are useful in explaining possible mechanisms of action, they may have limited application to humans as a result of interspecies differences in absorption, distribution, metabolism, and excretion. Additionally, the lack of standardization in noni content may also interfere with the search for valid conclusions. Therefore, human studies are necessary to determine what precise effects noni juice may have on human health. These studies should also provide information on phytochemical composition as noni fruit varies considerably according to region; concurrently, disparities exist in both the ingredients and processing methods used to produce various commercial noni juice products [21]. Accordingly, this review summarizes published human intervention studies involving primarily noni fruit juice (including mixed noni juice beverages) and discusses mechanisms of action that may explain the health benefits described in ethnobotanical studies. Also considered are the general limitations on the applicability of these human studies to noni juice products.

## 2. Clinical Safety Study

A 28-day clinical trial with 96 healthy adult volunteers was conducted to evaluate the safety of daily ingestion of Tahitian Noni^®^ Juice (TNJ), a pasteurized mixed noni juice from French Polynesia [22]. TNJ is composed of noni fruit juice from puree (89%) that is mixed with grape (*Vitis vinifera*) and blueberry (*Vaccinium corymbosum*) juices with an undisclosed proprietary final percentage of noni juice concentration [23]. The volunteers were divided equally into four dose groups: 0 mL (placebo), 30 mL, 300 mL, or 750 mL. Comprehensive hematology, biochemistry, and urinalysis tests were completed with each volunteer at the start of the trial as well as at week 2, week 4 (end of TNJ ingestion), and during a two-week follow-up (week 6). Electrocardiography tests were performed with each subject during a pre-study screening and at week six. Hematological measurements included hemoglobin, hematocrit, mean cell volume, red cell count, prothrombin time, activated partial thrombin time, total and differential white cell count (basophils, eosinophils, lymphocytes, monocytes, and neutrophils), and platelet count. Biochemistry analysis included alkaline phosphatase, alanine aminotransferase, aspartate aminotransferase, total bilirubin, lipids (LDL, HDL, cholesterol, triglycerides), creatine kinase, creatinine, gamma–glutamyl transferase, glucose, total protein, and uric acid. Urinalysis involved semi-quantitative analysis for leucocytes, nitrite, urobilinogen, protein, blood, ketones, bilirubin, glucose, pH, and specific gravity. Blood pressure, heart rate, weight, and adverse events were also recorded also at baseline and weeks 2, 4, and 6.

Test results revealed that there were no adverse dose-related effects from TNJ ingestion. During the entire study, no clinically significant differences were observed in any of the measurements between the placebo and TNJ groups, apart from a reduction between 20% and 50% of total adverse events (i.e., headache and other body aches) in the TNJ groups compared with the placebo group. The lower adverse event rates in the TNJ groups suggest possible health benefits from noni. The results of this study indicate that drinking up to 750 mL TNJ each day is safe.

## 3. Antioxidant and DNA Protection

A series of double-blind, placebo-controlled, 30-day intervention studies involving cigarette smokers has demonstrated the substantial antioxidant activity of a mixed noni juice (TNJ). The first of these involved 285 adult volunteers who smoked more than 20 cigarettes per day [24]. The participants were assigned randomly to a placebo, 29.5 mL TNJ/day, or a 118 mL TNJ/day group. The placebo was composed of grape and blueberry juices with a cheese flavor added to mimic the noni flavor. Blood plasma levels of superoxide anion radicals (SAR) and lipid hydroperoxide (LOOH) levels were measured at the beginning and end of the trial with participants continuing to smoke throughout the trial. TNJ ingestion reduced average plasma SAR by 26.9% and 30.8% in the 29.5 and 118 mL groups, respectively. Average LOOH levels in the 29.5 mL group were reduced by 24.5% while the 118 mL group experienced a 27.3% reduction. No significant reductions in SAR or LOOH levels occurred in the placebo group. Despite the presence of high antioxidant fruit juice in the placebo, the inhalation of large quantities of tobacco smoke resulted in more oxidative stress than could be overcome with grape and blueberry juices alone. Therefore, the SAR and LOOH reduction was attributable to the antioxidant properties, whether direct or indirect, of noni juice alone or in combination with the other ingredients.

The second smoker study was completed with 203 subjects [25]. The study intervention was the same as that mentioned above. However, in this study, peripheral blood lymphocytes (PBLs) were isolated from pre-and post-trial whole blood samples and evaluated for the degree of aromatic DNA adduct formation using a ^32^P-postlabeling assay. By the end of the 30-day study period, average aromatic DNA adduct levels dropped by 44.9% (*p* < 0.001) among those drinking either one fluid ounce (29.5 mL) or four fluid ounces (118 mL) of TNJ per day. There were no significant differences in gender-specific responses. But at the lower dose, males experienced a greater decrease in aromatic DNA adducts than females (56.1% vs. 43.1% reduction, respectively). No adverse effects were observed in this trial. 

Another randomized, double-blind, placebo-controlled study was completed with 245 heavy cigarette smokers [26]. This trial measured changes to lipid peroxidation–derived DNA adducts after 30 days of mixed noni juice beverage ingestion. Participants in this study were assigned to the same dose groups with the same placebo and TNJ that were used in the antioxidant trial. Again, a ^32^P-postlabeling assay was used following isolation of DNA from PBLs. The previous DNA protection study measured aromatic DNA adducts which were more likely to be formed by direct reactions with chemicals in cigarette smoke or their immediate metabolites. In this second study, measurements were made specifically of DNA adducts resulting from cigarette smoke-induced oxidative stress and consequent lipid peroxidation. At the completion of the trial period, the placebo group did not experience any reduction in DNA damage. However, the lipid peroxidation–derived DNA adduct levels in those who drank TNJ declined significantly by 46.9% to 57.4%. This effect is consistent with both previous studies where reactive oxygen species and aromatic DNA-adduct concentrations were significantly reduced.

## 4. Blood Lipid Normalization, High Sensitivity C-Reactive Protein (hs-CRP) and Homocysteine Reduction

As with the antioxidant and DNA adduct studies, adult smokers provided further insight into the effect of 30 days of TNJ ingestion on blood lipids and hs-CRP. In a randomized, double blind, placebo-controlled clinical trial with 132 volunteers [27], heavy smokers (≥20 cigarettes/day) were chosen as subjects because smoking has been reported to increase blood lipids, systemic inflammation, and serum homocysteine [28,29,30]. Pre- and post-study serum cholesterol, triglyceride, low density lipoprotein cholesterol (LDL), high density lipoprotein cholesterol (HDL), hs-CRP, and homocysteine were measured for all participants who continued smoking during the trial. In this study, the dose groups were the same as in the previously discussed smoker studies.

After drinking TNJ for 30 days, the placebo group had no significant changes in any measurements. But among those who drank TNJ, there were significant declines in average hs-CRP (15.2%) and homocysteine (23.9%) with an increase in HDL (from 49 to 57 mg/dL). Mean total cholesterol, LDL, and triglycerides were also decreased among those in the TNJ groups. However, the degree of change was dependent on initial (pre-trial) values. Larger declines in mean values were associated with greater initial total cholesterol, LDL, or triglycerides levels. For example, decreases in average total cholesterol in the low (190–219 mg/dL), middle (220–299 mg/dL), and high (>300 mg/dL) baseline strata of the 29.5 mL group were 12.1, 17.3, and 36.4%, respectively. No participants in either TNJ group experienced any changes that resulted in below normal reference values for individual blood lipid levels. As such, the changes experienced were towards or remained within normal healthy ranges. These results indicate that TNJ helped normalized lipid levels in heavy smokers.

A second, although smaller, study examined the cholesterol lowering activity of TNJ in nonsmokers [31]. This open-label pilot study involved older adults (>40 years) who had normal or borderline high cholesterol levels and were not taking cholesterol-lowering medication nor drinking alcohol. Each participant drank two fluid ounces (59 mL) of TNJ twice per day for 30 days. No other changes to lifestyle habits were made during the trial period. There was no significant difference between pre- and post-trial total cholesterol, HDL, and LDL levels, or TC/HDL ratio. These results suggest that the cholesterol modulating effect of TNJ in heavy smokers is likely a result of its ability to protect against cigarette smoke-induced dyslipidemia via antioxidant activity.

## 5. Improvement of Joint Pain and Mobility

Topical treatment of pain and bruising is one of the most common uses of the noni plant in tropical alternative medicine. Some animal studies suggest that noni possesses possible anti-inflammatory activity [32,33,34]. In fact, one of the traditional names for noni in the Caribbean was “pain killer” [35]. Among the published anti-nociceptive and anti-inflammatory studies of noni juice are two open-label clinical trials that demonstrate potential joint health benefits. The earlier of these two trials reported pain reduction and improved range of motion (flexion, extension, lateral flexion, and rotation) in patients suffering from cervical spondylosis after four weeks of ingesting 15 mL TNJ every morning and evening [36]. This trial enrolled 90 patients who were assigned to one of three treatment groups: standard physiotherapy alone, TNJ alone, and combined treatment (physiotherapy plus TNJ). Pre-and post-treatment measurements of pain intensity and neck flexibility (cervical range of motion) were compared within and among the treatment groups. At the start of the trial, all subjects in the TNJ alone group fell within the 5–7 (moderate to severe) pain intensity range. By the end of the study, the pain intensity range of this group had decreased to 0–4 (none to very moderate), with complete relief of neck pain in 60 percent of patients. The physiotherapy alone and combined treatment groups also experienced significant reduction in pain symptoms, with the combined treatment group experiencing the greatest reduction in pain intensity. Range of motion improved among all three treatment groups by the end of the trial period. For example, mean lateral flexion and rotation approximately doubled in the TNJ alone and physiotherapy alone groups. Although improvements in the TNJ alone group were no different than those of the standard physiotherapy alone group, a significantly greater improvement occurred in the combined treatment group.

A second joint health trial of TNJ involved osteoarthritis patients [37]. In this open-label intervention study, 82 volunteers drank three fluid ounces (88.5 mL) of TNJ (1 ounce before breakfast, 1 ounce before lunch, and 1 ounce before bedtime) every day. Those enrolled in the study were adults (40–75 years), had an X-ray diagnosis of osteoarthritis of the hip or knee, were not taking prescription medication for arthritis, and were willing to consume TNJ for 90 days. Blood samples were collected from the participants for clinical laboratory analysis at enrollment and after the 90-day intervention period. The Arthritis Impact Measurement Scales (AIMS2) were used to measure pre- and post-study pain/discomfort levels. The Short Form-36, version 2 (SF-36 V2) was used to measure pre- and post-study patient quality of life. 

By the end of the intervention period, significant improvements in mean quality of life measurements occurred. These included a reduction in the duration of arthritis pain, including a 23.7% decrease in the frequency (in days) of severe pain, and 16.4% decrease in pain severity. Patients also experienced an improved psychological state and mood and improved mobility. Patient satisfaction with personal health also increased by approximately 19%. As with previous studies, TNJ was well tolerated and appeared to be safe. No significant changes to liver or kidney functions occurred after three months of TNJ ingestion nor were there changes to blood glucose, total cholesterol, or triglyceride levels. It is important to note that since no placebo or control group was included in this trial, it is difficult to determine how much of the observed outcome was a result of the placebo effect. 

A double-blind placebo-controlled trial involving female university students suffering from dysmenorrhea was completed to evaluate the efficacy of a capsule containing 400 mg milled “noni herb powder”, calcium sulfate, gelatin, silica, and magnesium stearate [38]. Over the course of three menstrual cycles, noni capsule ingestion did not improve menstrual pain when compared with the placebo. However, there was some evidence of a significant decline in mean erythrocyte sedimentation rate (ESR) among those taking the noni capsule. This indicated some degree of anti-inflammatory activity, although not enough to affect pain symptoms. While this study did not involve noni fruit juice, it highlights issues regarding noni product identity and variation in potential efficacy. The product used in this study was identified as a “noni herb powder” with no indication as to the geographic origin, plant part used, or harvesting and processing conditions. As will be discussed in more detail, there is wide variability among nutrient and phytochemical compositions of commercial noni products [39]. These differences preclude assumptions that results from clinical trials of a specific noni product are applicable to other noni products. The two previously discussed trials involved the French Polynesian-derived noni juice blend (TNJ) used in the smoker studies. However, this latter trial evaluated a noni herb powder of relatively unknown identity. Therefore, the lack of significant pain reduction in the latter trial does not necessarily refute the pain reduction observations of the cervical spondylosis and osteoarthritis trials. However, it is interesting that all three trials did provide some evidence of anti-inflammatory activity.

## 6. Increased Physical Endurance

Some Pacific Islanders believed that ingesting ripe noni fruit invigorated the body during long fishing trips and ocean voyages [40]. Three clinical trials have been completed which lend some credence to this use. The first of these was a 21-day, placebo-controlled intervention study involving 40 highly trained middle and long-distance runners [41]. For three weeks, these athletes drank 100 mL TNJ or a blackberry juice placebo twice per day. The athletes participated in pre- and post-intervention time-to-fatigue treadmill tests during which the workload increased every minute. Pre-and post-intervention medical examinations (including electrocardiogram, heart rate, and blood pressure) and blood tests were also performed (including total protein, urea, glucose, hemoglobin, and lactate). The TNJ group had a 21% increase in average time-to-fatigue with an accompanying 25% decrease in average blood chemiluminescence (a marker of lipid oxidation), whereas no changes occurred in the placebo group. An accompanying study did not reveal the presence of any substances banned for athletic competition (including beta-blockers, diuretics, narcotics, anabolic steroids, stimulants, or masking agents). Therefore, it appears that the increase in physical endurance is associated with the antioxidant activity of TNJ.

A second similar trial was completed but with some modifications [42]. This trial equally divided 46 university athletes as well as 14 non-athlete controls into a TNJ group and a blackberry juice group. For 30 days, these athletes drank 100 mL TNJ or blackberry juice twice per day. This trial also included pre- and post-intervention time-to-fatigue treadmill tests, with accompanying blood analyses. The TNJ group experienced a significant decrease in mean serum creatine kinase (CK) concentration, while no such decline occurred in the blackberry juice group. This finding suggests that the improvement in endurance is a result of the ability of TNJ to mitigate exercise-induced muscle tissue damage. This protective effect likely involves the antioxidant properties of noni, as CK concentration increased along with oxidative stress markers in athletes undergoing intensive training [43]. Indeed, intense exercise causes increases in free radical production and inflammation [44] both of which are inhibited by TNJ. 

Noni juice may also have an influence on oxygen uptake during physical exertion. Semi-professional male cyclists were enrolled in a double-blind, placebo-controlled, randomized study to evaluate the effect of TNJ on aerobic fitness [45]. Volunteers drank either 120 mL of TNJ or a placebo every day for 14 days. At the beginning of the trial and after two weeks, volunteers completed exercise tests on a cycle ergometer (with 30 watt/min workload increase) to the point of exhaustion, electrocardiographic alterations, or to the theoretical maximal heart rate. Oxygen uptake was measured by spirometry. Increases in the average maximal oxygen uptake, VO_2_ max, and in oxygen uptake at 50-watt workload occurred in the TNJ group. TNJ does not contain any performance enhancing substances.

## 7. Increased Immune Activity

Smoking suppresses immune function [46]. Exposure to tobacco smoke is a risk factor for various bacterial and viral infections [47,48]. A smoking habit can have a significant negative impact of peripheral blood lymphocyte subsets and their function [49,50]. The ability of noni juice to protect lymphocyte DNA damage, as discussed above, may have a positive effect on the immune function of smokers. But noni juice may have an influence on immune function that goes beyond the protection of cells involved in the adaptive immune system. For example, the feeding of French Polynesian noni fruit puree to newborn cattle (Holstein bull calves) for the first two weeks of life significantly increased whole blood phagocytic activity against *E. coli* and *Staphylococcus epidermis* [51]. A follow-up study in Holstein bull calves revealed that noni puree supplementation for the first three weeks of life also reduced all required medical treatments by 54%, with a 61% reduction in respiratory treatments and a 52% reduction in gastrointestinal treatments [52]. The immune-modulating activity of noni juice was also observed in mice where TNJ consumption for 15 days resulted in the inhibition of mitogen-stimulated interleukin-4 production in splenocytes and peritoneal exudate cells [53]. This treatment also significantly increased interferon-gamma (IFN-γ) production. As IFN-γ is involved in macrophage activation, these results agree with the increase in phagocytic activity seen in newborn calves.

To verify the immune-modulating potential of noni juice in humans, an intervention study was conducted with 12 healthy adult volunteers for eight weeks, during which each consumed 330 mL of TNJ daily [54]. Vital signs, serum malondialdehyde (MDA) concentration, interleukin 2 (IL-2) concentration, and ex vivo natural killer-cell (NK) activity were measured before and after the intervention period. There was no change in vital signs during the study. However, there was a significant decrease in average MDA levels over the course of the trial due to reduced oxidative stress. On the other hand, mean IL-2 concentration and NK activity both increased by approximately 30%. The findings of this pilot study revealed TNJ’s potential for supporting immune function, along with concurrent antioxidant activity, in a healthy population.

Notably, the antioxidant properties of TNJ likely contribute to its immunomodulation activity as there is a documented connection between oxidative processes and the immune system. Reactive oxygen species (ROS) are involved in the modulation of immune function and serve as secondary messengers in cell signal transduction [55]. Multiple studies have demonstrated the ability of antioxidants, including those from fruits, to modulate immune responses [56,57,58,59]. Epidemiology studies have revealed associations between lower cancer incidence and diets rich in antioxidant nutrients, potentially stemming from antioxidant-induced improvement in immune function [60]. Antioxidant supplementation also improved immune function in the elderly and in other groups [61,62].

## 8. Control of Advanced Glycation End Products (AGEs) and Glycosylated Hemoglobin

Several in vitro studies have indicated that noni fruit may be helpful in controlling the formation of advanced glycation end products (AGEs) and may subsequently reduce their adverse effects. An extract from noni fruit collected in French Polynesia prevented AGE-induced reactive oxygen species generation in human umbilical vein endothelial cells [63]. This extract also inhibited the in vitro formation of glucose-human serum albumin, glucose-collagen, and glucose-keratin AGEs as well exhibited AGE-crosslink breaking activity [64]. Another noni fruit extract inhibited the development of glucose-induced bovine eye lens opacity [65]. 

The antiglycation potential of noni fruit juice was further demonstrated in an eight-week open-label intervention study and in a cross-sectional population study [66]. Both studies were conducted within the context of iridoid content, as iridoids are major phytochemicals in noni fruit and are well known for their anti-AGE biological activities [67,68]. Both studies utilized skin autofluorescence as a marker for AGE accumulation in the body [69]. The eight-week intervention study measured changes in the skin autofluorescence of 34 adults who daily consumed a mixed noni juice beverage similar to TNJ, TruAge^®^ Max (Morinda, Inc., American Fork, UT, USA). TruAge^®^ Max (MAX) is also composed of noni fruit puree but is mixed with cornelian cherry (*Cornus officinalis and Cornus mas*) juices, and olive (*Olea europaea*) leaf extract. Overweight or obese, prehypertensive, or grade 1 hypertensive males and females with impaired fasting glucose and who were not using prescription medication were included in the trial. Previously published population reference values revealed that the average initial skin autofluorescence of this group was typical of healthy 44-year-old adults even though their average actual age was 40. As such, their AGE-associated age (ASA) was four years older than their average actual age. But eight weeks of MAX supplementation reduced the ASA of this group to 39 years, demonstrating a significant antiglycative effect. 

The cross-sectional population study included 3913 people from ten locations throughout Japan. During health education and promotion events, a questionnaire was used to collect demographic data and information on daily ingestion rates of mixed noni juice beverages (i.e., TNJ or MAX). Daily iridoid intake was calculated from the ingestion rates by applying the data obtained from chemical analyses of the beverages. As with the eight-week intervention study, skin autofluorescence was used as a marker for AGE burden in 2790 mixed noni juice consumers and 1123 controls (those who did not drink any noni juice beverages). Regression analysis revealed that mixed noni juice intake was associated with lower AGE levels with noni consumers having an average ASA that was 2.07 years less than the general population. Notably, for every mg of iridoids consumed, ASA decreased by 0.017 years. Among those who never smoked, the average ASA of mixed noni juice consumers was 3.52 years less than the general population. The significant anti-AGE activity of mixed noni juice was at least in part a result of its ability to induce antioxidant enzyme activity, specifically superoxide dismutase and catalase [70]. 

Recently, some Pacific Islanders have considered noni fruit to be helpful in controlling blood sugar levels [71]. In vitro and animal studies have provided some supporting evidence for this view [72,73,74,75]. Even so, data from human clinical trials is limited. A small eight-week human pilot study was conducted with Type 2 diabetes patients [76]. In this study, ingestion of 2 mL of TNJ per kg body weight, twice daily, was associated with improved blood glucose profiles and a significant reduction in glycosylated hemoglobin (HbA1c). It should be noted, however, that TNJ did not influence blood glucose levels in those without diabetes [22]. Therefore, the anti-diabetic property of TNJ appears to only involve the regulation of normal blood glucose level and is limited by feedback mechanisms, rather than involving the pharmacological pathways that are targets of conventional diabetes medications.

## 9. Weight Management and Mitigation of Osteoporosis, Hypertension, and Gingivitis

Currently, several Pacific Islanders believe that noni plant preparations are useful for weight management [71,77]. Multiple animal studies have demonstrated the anti-obesity potential of noni juice [78]. Two clinical trials have further investigated this potential. The first of these was a 12-week, open-label trial of a weight-loss program that included TNJ, daily calorie restriction, and exercise interventions [79]. By the end of the trial, all participants experienced weight loss with mean percent body fat decreasing by 8.91% (*p* < 0.0001). Five of 22 participants moved from overweight to the normal weight category while another five switched from obese to overweight. However, given the other interventions also included in the trial (i.e., exercise and calorie restriction), it is difficult to determine the degree to which noni juice contributed to this outcome. 

The second weight loss study included 90 grade 3 overweight (morbidly obese) adults who were divided into three treatment groups [80]. For six weeks, all participants followed a low calorie and low sodium diet. Participants in two separate mixed noni juice groups were assigned to drink either TNJ or MAX for the duration of the study. Those in the control group did not consume any noni juice beverages. Muscle mass loss was significantly lower in those drinking mixed noni juice beverages compared to the control group. Maintenance of weight loss throughout the study was greater in those drinking TNJ or MAX than in the control group as was reduction of waist circumference and body mass index. The preservation of active muscle cell mass seems to be a likely mechanism by which the improved weight loss was achieved by those in the TNJ and MAX groups. Protection of muscle tissue is also one mechanism responsible for improved endurance, as previously discussed.

A somewhat unexpected property of noni juice was its potential influence on osteoporosis and conductive hearing loss. A three-month placebo-controlled pilot study was conducted with post-menopausal women wherein participants drank either two fluid ounces (59 mL) of TNJ or placebo twice per day [81]. Before and after the study, the women completed the Short Form 36 (SF-36) Quality of Life Survey and provided urine samples for deoxypyridinoline analysis (a marker for bone turnover) as well as received hearing tests. Those who drank TNJ had slightly greater average deoxypyridinoline crosslinks, indicating increased bone resorption. This finding was consistent with the observed attenuation of hearing loss that also occurred in the TNJ group, as the transmission of sound vibrations by the bones of the middle ear is influenced by the severity of osteoporosis. These positive effects on bone health also accompanied improvements in several quality of life scores including mental health scores. 

Two additional small human pilot studies suggest that noni juice may also have a positive impact on hypertension and gingivitis. In a one-month open-label trial, hypertensive adults drank two fluid ounces (59 mL) of TNJ twice per day [82]. Pre- and post-trial diastolic and systolic blood pressure measurements were made in triplicate and compared. By the end of the study, all participants experienced reductions in systolic blood pressure with average diastolic and systolic readings decreasing from 83 to 76 mm Hg and from 144 to 132 mm Hg, respectively. Accompanying in vitro testing suggested that pure noni juice may have some effect on angiotensin converting enzyme (ACE) and angiotensin receptors (AR). However, this trial included a small number of participants (*n* = 10), and there was no effect on the blood pressure of normotensive participants in other clinical trials of TNJ. Therefore, ACE-inhibiting and AR-blocking activities are not likely to occur in real-life conditions. If noni juice does have an impact on high blood pressure, it may involve other mechanisms such as reduction of aortic SAR and the release of nitric oxide by macrophages [83]. These same antioxidant and anti-inflammatory mechanisms are reportedly responsible for the hypotensive activity of olive leaf extract [84,85]. 

To evaluate the influence of TNJ on oral health, eleven patients with moderate to severe gingivitis or periodontitis were enrolled in a four-week pilot study [86]. These volunteers were divided into a control group (*n* = 5) and a TNJ group. Those in the TNJ group rinsed their mouths for two minutes with 30 mL TNJ plus 30 mL water, followed by swallowing. This was done twice per day for four weeks. Bacterial samples were isolated from the oral cavity and gingival pouches. Papilla bleeding (PBI), plaque, and approximal plaque indices were also scored for each volunteer before and after the treatment period. Those in the TNJ group experienced a significant decline in PBI, especially when compared to the control group. Interestingly, TNJ had only weak bacteriostatic activity in vitro, and bacterial composition of patient oral cavities did not change significantly during the treatment phase. Therefore, the study authors concluded that the improvement in clinical outcomes in the TNJ group was likely a result of the anti-inflammatory properties of noni juice.

The human intervention studies described in this section as well as in the preceding sections are summarized in Table 1. The experimental designs employed have ranged from small pilot studies with few participants to double-blind placebo-controlled clinical trials involving hundreds of volunteers. 

## 10. Noni Extract Studies

There have been several clinical trials with various noni extracts. The phytochemical compositions of these extracts are considerably different than that of noni fruit juice. Therefore, the conclusions drawn from these studies may not be applicable to noni juice. However, they are briefly described in this review as they may provide some additional insight into the general health-promoting properties of the noni plant.

A phase 1 clinical trial of freeze-dried noni fruit capsules found no evidence of toxicity but did reveal dose-dependent improvements in physical functioning, pain, and fatigue in patients with progressing advanced cancer for which no standard treatment was available [87]. A randomized, placebo-controlled clinical trial with 100 adult patients revealed that an aqueous extract of dried whole noni fruit prevented postoperative nausea when ingested one hour before surgery [88]. Ingestion of an aqueous noni fruit extract increased ranitidine absorption in healthy adult volunteers, indicating a possible effect on gastrokinetic activity that might make it useful as a carminative and appetite-stimulating agent and in the relief of heartburn [89]. A noni fruit extract exhibited analgesic properties comparable to or better than ibuprofen in a randomized parallel clinical trial involving 51 patients who had undergone simple tooth extraction procedures [90]. The results of a three-month open-label longitudinal study demonstrated the potential anti-stress (adaptogenic) effect of an herbal mixture that included noni [91]. Human trials involving the topical application of noni extracts as well as noni fruit juice indicate anti-inflammatory, anti-acne, and anti-aging activities within the skin [92,93,94,95,96].

## 11. Discussion

Considerable evidence among the results of these human studies has demonstrated that noni juice has notable antioxidant activity, more than the other common fruit juices that were used in the placebos. It suggests that the antioxidant effects of TNJ are universal as it has been observed in heavy smokers, athletes, and in ordinary healthy people. This antioxidant activity may involve direct chemical reactions between phytochemicals and reactive oxygen species or may involve the induction of antioxidant enzyme systems. Examples of these two mechanisms were found in both in vitro and in vivo studies of noni juice.

TNJ was also evaluated for its in vitro antioxidant activity in the LOOH and tetrazolium nitroblue (TNB) assays [97]. The SAR scavenging activity of TNJ, as measured in the TNB assay, followed a linear positive dose-response. The SAR scavenging activity of 7 μL/mL TNJ was also compared to those of 13.3 μg/mL vitamin C, 13.3 μg/mL Pycnogenol^®^ (Twin Labortories, Inc., New York, NY, USA), and 22.2 μg/mL of grape seed powder, the latter three being well known for their antioxidant activity. The SAR-scavenging activity of TNJ was 2.8 times greater than vitamin C, 1.4 times greater than Pycnogenol^®^, and 1.1 times greater than grape seed powder. Notably, the total solid material in 7 μL TNJ was approximately 0.7 μg. Therefore, a much smaller amount of noni juice solids, 3 to 5% of the total weight of the other antioxidants exhibited greater SAR-scavenging activity. As with SAR scavenging activity, in vitro LOOH quenching activity followed a linear positive dose-response, revealing a consistent antioxidant action.

Ingestion of TNJ protected the livers of Sprague-Dawley (SD) rats exposed to carbon tetrachloride and resulted in decreased hepatotoxic lesions and significant reductions in serum alanine aminotransferase (ALT) and aspartate aminotransferase (AST) when compared to a placebo group [98]. Further, SAR and LOOH levels were significantly lower in the liver tissue of noni-fed rats [99]. Carbon tetrachloride (CCl_4_) causes extensive liver damage from lipid oxidation processes as a result of the formation of the trichloromethyl and trichloromethyl peroxide radicals during metabolism. These trichloromethyl radicals initiate lipid peroxidation of unsaturated fatty acids resulting in cytotoxic and genotoxic LOOH and other decomposition products as well as increased pro-inflammatory SAR production [100,101]. As such, the liver protective results in these in vivo studies suggest that noni juice may contain compounds that are capable of scavenging trichloromethyl radicals or subsequent peroxidation products.

Ingestion of TNJ by ICR mice for 30 days resulted in a dose-dependent decrease in blood MDA as well as dose-dependent increases in glutathione peroxidase and superoxide dismutase activities in the liver [102]. Feeding of deacetylasperulosidic acid (DAA), a major phytochemical constituent of noni juice, for seven days to Wistar rats resulted in a significant increase in enzymatic antioxidant activity. Further, a dose-dependent reduction in serum MDA concentration, a downstream oxidation product of SAR action, accompanied an increase in superoxide dismutase activity, suggesting that DAA ingestion increased catalase activity [70].

As previously discussed, the antioxidant properties of noni juice, likely, contribute to its immune modulating activity. This attribute may also be at least partially responsible for noni’s anti-inflammatory activity. The relationship among inflammation, the immune system, and reactive oxygen species (ROS) is well established in the scientific literature. Leukocytes release pro-inflammatory cytokines and ROS which, in turn, cause a “respiratory” or oxidative burst by NADPH oxidase. This leads to further inflammation as well as recruits and activates other leukocytes. This inflammatory process leads to tissue damage such as that occurring in certain types of muscle injury [103]. Antioxidants have been shown to suppress neutrophil chemotaxis (recruitment) and reactive oxygen intermediates [104,105]. Antioxidants also have an influence on cyclooxygenase-2 (COX-2) expression and activity. Reactive oxygen intermediates are involved in signaling pathways that lead to COX-2 expression in cells. Inhibition of COX-2 expression has been observed in vitro, in vivo, and ex vivo for a variety of radical scavengers [106,107,108]. As the rate of prostaglandin synthesis by cyclooxygenases is dependent on peroxide, antioxidants and enzymes that limit peroxide availability may inhibit cyclooxygenase activity [109,110]. Similarly, lipoxygenase is activated by ROS [111]. Therefore, antioxidants may exhibit anti-inflammatory activity by way of multiple mechanisms. 

The antioxidant mechanisms induced by noni juice appear to be involved, to some degree, in all of the effects reported in human interventions studies. It is very likely that this property of noni juice is central to its observed effects on physical fatigue, weight control, osteoporosis, hypertension, and gingivitis. Certainly, antioxidant action is a major mechanism by which noni juice controls AGE formation [68]. 

There are limitations in applying the results of the human studies discussed in this review to noni products in general. First, these noni juice studies were conducted on one commercial source. This source is noni fruit puree from French Polynesia, and the safety and efficacy of other sources may not be the same. As briefly mentioned above, there is wide variability among the nutrient and phytochemical compositions of commercial noni products. An analysis of 177 commercial noni juice products revealed large differences among the mineral content of these products [39]. One reason for this is the inclusion of different ingredients in these juice products. Another reason is the use of varying amounts of different types of noni materials. From a safety perspective, this variability is significant. An example of this is provided in a liver injury case involving a 14-year old boy who had been ingesting what was purportedly a noni juice dietary supplement [112]. However, subsequent chemical analysis of this supplement revealed that it contained less than 1% noni juice as well as included other ingredients that were not declared on the product label. Ingredients other than noni were responsible for the liver injury [113]. Another example is a product that was marketed as a noni fruit extract but was devoid of any of the well-known chemical markers of noni fruit. After evaluation in a reproductive toxicity test, this counterfeit noni extract was reported to be potentially less safe than authentic noni fruit that had been evaluated in several other reproductive toxicity tests [114,115]. A third example is a case of liver injury from a noni product of unknown identity that was purchased from a local market in Ecuador and brought to Europe for personal use [116]. This product was not produced with the same quality controls that ensure an authentic and safe noni juice product and was not a product approved for use by the European Union.

These particular instances of product adulteration are a warning that a label declaration of “noni juice” does not guarantee that a product is either safe or authentic. Authentic noni juice made from noni fruit puree from French Polynesia (TNJ) has been subject to thorough safety assessments by European Union officials. Consumption of this source of noni fruit juice has been determined to be safe as have ingredients derived from the same source [117,118]. Nutrient and chemical specifications for these ingredients were set in place to provide an identity for what has been demonstrated to be safe [119]. When noni fruit is processed into puree, the seeds and skin are removed. There are several consequences to this in terms of phytochemical content. Among these is an increase in the safety profile due to the prevention of possible anthraquinone contamination [120]. This is one reason why processing of noni fruit into puree is a recommended and approved method.

Differences in harvesting and processing methods for noni fruit also have an impact on potential efficacy. For example, the degree of ripeness at harvest and subsequent post-harvest aging may influence antioxidant activity as well as vitamin C and phenolic compound content [121,122]. Also, changes that occur during long periods of fermentation include significant losses in antioxidant activity [123]. On the other hand, production of noni fruit puree does not seem to significantly affect deacetylasperulosidic acid, scopoletin, or vitamin C content [67,124]. 

Geographical, or environmental, factors (soil, sunlight, temperature, precipitation, etc.) may significantly influence noni fruit composition. Scopoletin, rutin, quercetin, and 5,15-dimethylmorindol were detected in greatly varying concentrations in noni fruits and noni fruit juice products sourced from 13 different nations located in the Caribbean, Central America, the Central Pacific, the South Pacific, and Asia [125]. Iridoid content was also substantially different among samples of noni fruit collected from French Polynesia, Tonga, the Dominican Republic, Okinawa, Thailand and Hawaii, with French Polynesia containing the highest concentration and the Dominican Republic containing the lowest [67]. Principle component analysis of commercial noni juice products has also revealed regionally distinct phytochemical profiles [126]. Further, iridoid glucoside, scopoletin, rutin, and fatty acid glucoside concentrations were considerably different among various samples of noni fruits, powdered fruit capsules, noni juices, and mixed noni fruit juices. Noniosides B and C as well as scopoletin were even absent in some samples of commercial noni juice [127].

## 12. Conclusions

The weight of evidence obtained from human studies point to noni juice’s greater antioxidant activity than the other fruit juices that served as placebos. It is this activity and its interaction with the immune system and inflammation pathways that may account for much of the observed health benefits of noni juice. These health benefits may include protection against tobacco smoke toxicities—including DNA protection, normalization of blood lipids, control of systemic inflammation, and reduction of homocysteine—improvement of joint pain and mobility, increased physical endurance, increased immune activity, control of AGE accumulation, weight management, maintenance of bone health in women, control of blood pressure, and improved gum health. 

The existing evidence does have some general limitations in its application to noni juice products. Geographical factors, along with post-growth factors (harvesting, storage, transportation, processing, and formulation) produce commercial noni juice products with different phytochemical and nutrient profiles. Differences in phytochemical profiles will likely result in variations in biological activity [128]. As such, conclusions drawn from the human clinical trials discussed in this review may be limited to a single source of noni juice. Noni juice products from other sources are likely to have somewhat different toxicological and pharmacological profiles.

## Figures and Tables

**Table 1:** Summary of human intervention studies of noni juice products.

Study Reference	Study Type	Study Population	Number of Subjects in Study	Treatment	Oral Doses	Duration	Main Outcomes
West et al. 2009 [22]	Double blind, placebo-controlled clinical safety study	Healthy adults	96	Mixed noni juice beverage (TNJ) *	0 mL (placebo), 30 mL, 300 mL, and 750 mL daily	28 days	No dose-related adverse events or effects on clinical chemistry and hematological measurements, urinalysis, electrocardiogram, blood pressure, heart rate, or body weight.
Wang et al. 2009 [24]	Double blind, placebo-controlled clinical trial	Heavy cigarette smokers (>20 cigarettes/day)	285	Mixed noni juice beverage (TNJ) *	0 mL (placebo), 29.5 mL, and 118 mL daily	30 days	26.9–30.8% reduction in mean plasma superoxide anion radicals (*p* < 0.001) and 24.5–27.3% mean reduction in plasma lipid hydroperoxides (*p* < 0.001).
Wang et al. 2009 [25]	Clinical trial, no placebo comparator reported	Heavy cigarette smokers (>20 cigarettes/day)	203	Mixed noni juice beverage (TNJ) *	0 mL (placebo), 29.5 mL, and 118 mL daily	30 days	44.9% average reduction in aromatic DNA adducts in peripheral blood lymphocytes (*p* < 0.001).
Wang et al. 2013 [26]	Double blind, placebo-controlled clinical trial	Heavy cigarette smokers (>20 cigarettes/day)	245	Mixed noni juice beverage (TNJ) *	0 mL (placebo), 29.5 mL, and 118 mL daily	30 days	20.3–25.6% reduction in mean total cholesterol (*p* < 0.05), 29.4–41.2% reduction in mean triglycerides (*p* < 0.05), 15.2% reduction in mean hs-CRP (*p* < 0.001), and 23.9% reduction in mean homocysteine (*p* < 0.05). Mean HDL increased from 49 to 57 mg/dL (*p* < 0.05).
Wang et al. 2012 [27]	Double blind, placebo-controlled clinical trial	Heavy cigarette smokers (>20 cigarettes/day)	132	Mixed noni juice beverage (TNJ) *	0 mL (placebo), 29.5 mL, and 118 mL daily	30 days	44.6–57.4% reduction in lipid peroxidation-derived DNA adducts in peripheral blood lymphocytes (*p* < 0.001).
Palu et al. 2012 [31]	Open-label pilot study	Adult (>40 years age) non-smokers with normal to mildly elevated cholesterol	10	Mixed noni juice beverage (TNJ) *	59 mL twice per day	30 days	No significant difference between pre- and post-trial total cholesterol, HDL, or LDL levels.
Akinbo et al. 2006 [36]	Open-label, conventional treatment-controlled trial	Cervical spondylosis patients	90	Mixed noni juice beverage (TNJ) *	15 mL twice per day	4 weeks	60% of patients in noni group experienced pain relief and improvement in range of motion. Efficacy rate was not significantly different from conventional treatment (*p* > 0.05)
Wang et al. 2011 [37]	Open-label trial	Osteoarthritis patients	82	Mixed noni juice beverage (TNJ) *	3 fluid ounces (88.5 mL) daily	90 days	23.7% reduction in frequency (in days) of severe pain (*p* < 0.05) and 16.4% decrease in pain severity (*p* < 0.05). Improved psychological state and mood and improved mobility (*p* < 0.001).
Palu et al. 2008 [41]	Placebo-controlled clinical trial	Highly trained athletes (middle and long-distance runners)	40	Mixed noni juice beverage (TNJ) *	100 mL twice per day	21 days	21% increase in mean time-to-fatigue (*p* < 0.05). 25% decrease in mean blood chemiluminescence/oxidation (*p* < 0.05).
Anugweje et al. 2012 [42]	Placebo-controlled clinical trial	University athletes	46	Mixed noni juice beverage (TNJ) *	100 mL twice per day	30 days	Significant reduction in mean serum creatine kinase (from 209.8 to 148.1 IU/L, *p* = 0.001) after time-to-fatigue tests.
West et al. 2013 [45]	Double blind, placebo-controlled clinical trial	Semi-professional cyclists	20	Mixed noni juice beverage (TNJ) *	120 mL daily	14 days	Increases in oxygen uptake at 50-watt workload (from 15.2 to 17.4 mL/kg/min, *p* = 0.005) and VO_2_ max (from 51.5 to 55.0 mL/kg/min, *P* = 0.009)
Ma et al. 2008 [54]	Open-label pilot study	Healthy adults	12	Mixed noni juice beverage (TNJ) *	300 mL daily	8 weeks	Mean serum malondialdehyde levels declined from 4.81 to 3.90 nmol/mL (*p* < 0.01), mean serum IL-2 increased from 52.5 to 69.2 pg/mL (*p* < 0.05), and mean natural killer cell activity increased from 27.7 to 36.0% (*p* < 0.05).
West et al. 2014 ^§^ [66]	Open-label pilot study	Overweight or obese adults with grade 1 hypertension and impaired fasting glucose	34	Mixed noni juice beverage (MAX) ^†^	60 to 240 mL daily	8 weeks	Decrease in mean skin autofluorescence units, a measurement of advanced glycation end products (A.G.E.s), from 1.89 to 1.77 units (*p* < 0.05).
Palu et al. 2011 [79]	Open-label pilot study	Adults with a body mass index greater than 25	22	Mixed noni juice beverage ^‡^, exercise intervention, calorie restriction, and other dietary supplements	≥30 mL daily	12 weeks	Every participant in the trial experienced weight loss. Mean percent body fat decreased by 8.91% (*p* < 0.0001). It is difficult to determine the degree to which noni juice contributed to this outcome.
Bogdanov et al. 2015 [80]	Open-label prospective study in 3 parallel groups	Obese adults (grade 3)	90	Low sodium and calorie restricted diet, as well as 2 different mixed noni juice beverages (TNJ, MAX) *^,†^ (each used by a different group)	60 mL twice per day	42 days	After 6 weeks of calorie restriction, the average loss of lean muscle mass was 3.1–4.1% in the two noni groups, whereas it was 8.5% in the control group (*p* = 0.0051). Maintenance of weight loss throughout the 6-week period was greater in the noni groups than in the calorie restriction only group.
Langford et al. 2004 [81]	Placebo-controlled pilot study	Post-menopausal women	8	Mixed noni juice beverage (TNJ) *	2 fluid ounces (59 mL) twice per day	3 months	Improved mental health score in SF-36 clinical survey (*p* = 0.05). Trend of slight increase in mean urinary deoxypyridinoline concentration. Attenuated hearing loss at 8000 Hz compared to the placebo group (*p* = 0.05).
Palu et al. 2008 [82]	Open-label pilot study	Hypertensive adults	10	Mixed noni juice beverage (TNJ) *	2 fluid ounces (59 mL) twice per day	4 weeks	Systolic blood pressure (BP) was reduced in all participants. Diastolic BP was reduced in nine participants. Mean systolic and diastolic BPs were reduced from 144 to 132 mmHg and from 83 to 76 mmHg, respectively.
Glang et al. 2013 [86]	Open-label pilot study with a no-treatment control group	Adults with moderate to severe gingivitis/periodontitis	11	Mixed noni juice beverage (TNJ) *	30 mL twice per day	4 weeks	Decline in mean papilla bleeding index (from 2.25 to 1.01) was significantly greater than that of the control group (*p* = 0.01).

* Tahitian Noni^®^ Juice (TNJ, manufactured by Morinda, Inc., American Fork, UT, USA); contains noni juice from puree (89%) as well as grape (*Vitis vinifera*) and blueberry (*Vaccinium corymbosum*) juices. ^†^ TruAge Max (MAX, manufactured by Morinda, Inc., American Fork, UT, USA); contains noni juice from puree, olive (*Olea europaea*) leaf extract and cornelian cherry (*Cornus officinalis and Cornus mas*) juices. ^‡^ Tahitian Noni^®^ Original Bioactive Beverage (manufactured by Morinda, Inc. American Fork, UT, USA); contains noni juice from puree (89%) as well as grape and blueberry juices. ^§^ A cross-sectional population study (*n* = 3913 Japanese volunteers) of the anti-AGE effect of mixed noni juice beverage ingestion, was not included in this table, as it is not an intervention study.
